# Strength of the porcine proximal femoral epiphyseal plate: the effect of different loading directions and the role of the perichondrial fibrocartilaginous complex and epiphyseal tubercle – an experimental biomechanical study

**DOI:** 10.1186/s40634-014-0004-y

**Published:** 2014-06-26

**Authors:** Páll Sigurgeir Jónasson, Lars Ekström, Anna Swärd, Mikael Sansone, Mattias Ahldén, Jón Karlsson, Adad Baranto

**Affiliations:** Department of Orthopaedics, Institute of Clinical Sciences at Sahlgrenska Academy, University of Gothenburg and Sahlgrenska University Hospital, Gothenburg, Sweden; Orthocenter/IFK-KLINIKEN, Sports Medicine Clinic, Gothenburg, Sweden; Östersund Hospital, Östersund, Sweden

**Keywords:** Hip, Load, Epiphysiolysis, Epiphyseal plate, Porcine, Epiphyseal tubercle, Perichondrial fibrocartilaginous complex

## Abstract

**Background:**

The high loads on adolescent athletes’ musculoskeletal system are known to cause morphological and degenerative changes in bone, intervertebral discs and joints. It has been suggested that the cam deformity of the proximal femoral head originates from a subclinical slipped capital femoral epiphysis (SCFE) as a result of non-physiological loading. The perichondrial fibrocartilaginous complex (PFC) and the epiphyseal tubercle are believed to stabilise the proximal femoral epiphysis, but their role is still unclear. The aim of the present study was to develop an experimental, biomechanical model to evaluate the strength of the porcine proximal femoral epiphysis in different loading directions and, furthermore, to investigate the stabilising role of the PFC and the epiphyseal tubercle.

**Methods:**

A descriptive laboratory study. An *in-vitro* model was developed and nine young (5 months) porcine proximal femoral epiphyses were loaded to failure; three in the anterior-posterior direction, three in the lateral-medial direction and three in the vertical direction. The injured proximal femoral epiphyses were then examined both macroscopically and histologically.

**Results:**

Anterior and lateral loading of the proximal femoral epiphysis resulted in failure of the epiphyseal plate, while vertical loading resulted in a fracture epiphyseolysis. The epiphysis was weakest when exposed to a lateral load and strongest when exposed to a vertical load. Despite histological epiphyseolysis, the PFC was intact in 15 of 27 (56%) slices. In histological examinations, the epiphyseal tubercle appears to halt the slide of the epiphysis.

**Conclusions:**

We have developed an experimental, biomechanical model to measure the strength of the proximal femoral epiphyseal plate in different loading directions. The strength of the proximal femur was weakest through the epiphyseal plate. The epiphysis was weakest when exposed to a lateral load and strongest when exposed to a vertical load. The epiphyseal tubercle and the PFC stabilise the epiphysis when the epiphyseal plate is damaged. The findings in the present study indicate that overloading the hips in growing individuals can disrupt the epiphyseal plate. These findings may have implications when it comes to understanding the pathogenesis of cam deformity of the hip.

## Background

The skeleton of the growing individual is susceptible to mechanical injuries, but the growing bone is also capable of adapting to varying stresses [1]. Some physiological load is needed for normal bone growth, [2] but exceeding this physiological load can lead to unwanted morphological changes and different pathologies of the bone and joints [3,4].

Adolescent athletes in sports where high demands are imposed on the back have been shown to have a high frequency of abnormalities in the vertebral endplates, apophyseal ring and intervertebral discs [5,6]. A systematic review of sports-related growth plate injuries reported similar results for the upper and lower extremities [3].

The cam deformity is a non-spherical extension of the femoral head at the antero-superior head-neck junction of the proximal femur. It is more common in adolescent athletes, participating in high-load sports, compared with an age-matched control group [7] and it is thought to be a frequent cause of hip pain in athletes [8]. The etiology of the cam deformity is not known. One suggested explanation is that it is caused by a subclinical epiphysiolysis of the proximal femoral epiphysis [9–11].

In 1976, Chung et al. reported that the perichondrial fibrocartilaginous complex (PFC) was an important stabiliser of the human epiphyseal plate. When the PFC was excised, the shear strength of the plate decreased significantly [12]. Other researchers have proposed that a defect in the PFC or thinning of the periosteum may precipitate subclinical slipped capital femoral epiphysis (SCFE) [13,14]. The epiphyseal tubercle is also believed to stabilise the proximal femoral epiphyseal plate. It is a tuberosity, eccentrically and inferiorly placed on the capital epiphysis, projecting into an appropriately shaped socket in the metaphysis. The tubercle is believed to hold the epiphysis in place in a chronic SCFE [15–17].

During routine daily activities, the hip joint is mainly loaded vertically [18–21]. The way the load is distributed in the hip joint in deep flexion or during sport is largely unknown.

The purpose of the present study was to create a porcine model in order to estimate the difference in strength of the proximal femoral epiphysis in relation to different loading directions. Furthermore, the aim was to explore the stabilising roles of the PFC and epiphyseal tubercle, utilising a histological analysis.

The hypothesis was that the epiphyseal plate would be the weakest link in the proximal femur and that its strength would be greatest during vertical loading.

## Methods

### Experimental animals and procedures

The proximal femur from nine young (5 months) porcine hips were dissected and cleaned of soft tissue, muscle and capsular attachments. The porcine subjects were male domestic pigs, obtained from the local abattoir, with a median weight of 90 kg (range 85–95). The diameter of the femoral heads was measured in the coronal and sagittal planes with a measurement error of less than 1 mm using a slide caliper [22].

### Mechanical test procedures

Nine femurs were used in pilot tests of the model in order to establish a correct fixation method; the remaining nine femurs were used for the study.

The prepared proximal femurs were fixed with cement in a metal fixture and attached to the actuator of a servo hydraulic universal testing machine (MTS Test Star, Minneapolis, MN, USA). A custom-made aluminium rod with a small plastic cup (25 mm in diameter), contoured to the shape of the femoral head, was attached to the upper crosshead of the testing machine. The deformation control mode was set at a rate of 0.05 mm/sec. Force and deformation were recorded continuously and failure was defined as a sudden decrease in force, which could be seen on the force/deformation diagram on the computer. Furthermore the failure was visual and audible to the researchers (the first and second authors) in all test cases. Loading was stopped immediately after the first sign of failure.

**Figure 1:**
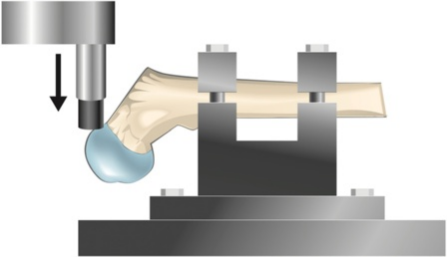
**Set-up for lateral loading of the specimens.** The major trochanter was removed to facilitate access for the loading rod.

**Figure 2:**
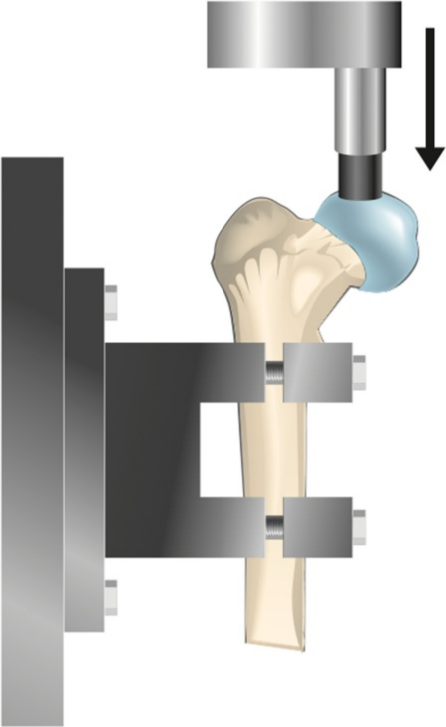
**Set-up for vertical loading of the specimens.**

**Figure 3:**
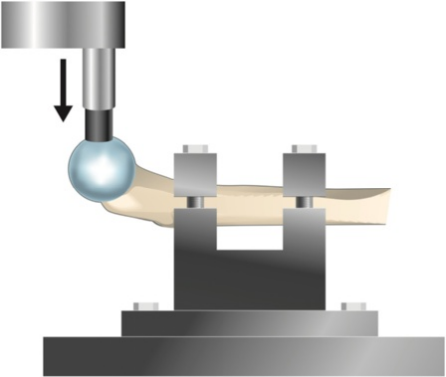
**Set-up for anterior loading of the specimens.**

The physeal line was clearly visible on the surface of the femoral head and was used as a guide for the placement of the plastic cup, which was proximally to the line. The proximal epiphysis was then loaded to failure in one of three directions. Three femurs were loaded from the lateral side in the coronal plane, three femurs were loaded from the anterior side in the sagittal plane and three femurs were loaded vertically (Figures 1, 2 and 3). On those loaded from the lateral side, the major trochanter was removed to facilitate access for the loading rod with the plastic cup. After failure, the size of the footprint left by the plastic cup on the femoral head and its distance from the physeal line were measured. Any signs of epiphyseolysis or fracture, visual or palpable, were registered if present.

### Macroscopic and histological examinations

The specimens were then stored in a −20°C freezer until completely frozen. When frozen, they were sawn into four equally thick primary slices in the loading plane using a bandsaw. The slices were decalcified, dehydrated, fixed in paraffin and cut in 4 μm slices using a microtome. Four slices from each specimen were then stained with hematoxylin-eosin and alcian blue solution. The histological slices were examined microscopically for injuries by the first and the second author (an orthopedic surgeon and a research engineer respectively).

The histological presence of epiphyseolysis, the percentage of the epiphyseal plate that was damaged, the epiphyseal zone that was damaged and a Salter-Harris classification were registered for each slice. The integrity of the PFC and the presence of the epiphyseal tubercle were also noted and registered.

## Results

### Anterior load

**Figure 4:**
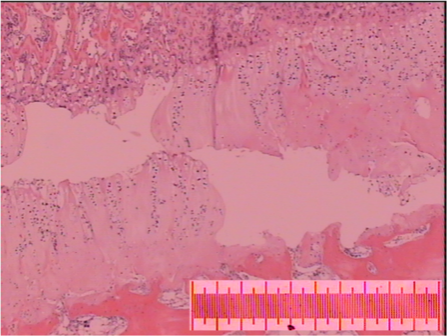
**Photomicrograph showing damaged epiphyseal plate.** In the anterior and lateral loaded specimens, the damage wandered between the resting and ossifying zones. Sample stained with hematoxylin-eosin solution. Magnification X25. Scale bar equals 1 mm.

**Figure 5:**
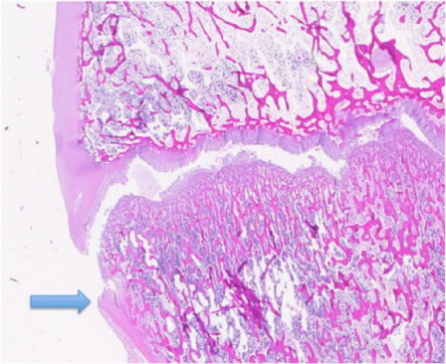
**Epiphysiolysis with a damaged PFC (arrow).** Sample stained with hematoxylin-eosin solution. Scanned slide, no magnification.

**Figure 6:**
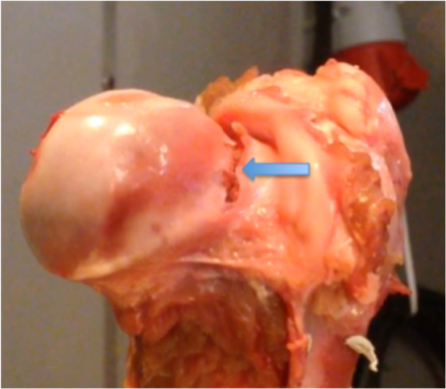
**A small cleavage (arrow) over the epiphyseal line can be seen after failure.**

Imposing an anterior load on all three specimens resulted in failure at a median load of 1,750 N (range 1,679-1,756). On histological examination, subtotal epiphyseolysis was present in two specimens and total epiphyseolysis was present in one specimen. The damage wandered erratically between the resting and the ossifying zones of the epiphyseal line (Figure 4). PFC damage could not be assessed in one slice and was seen on only one slice of the remaining eleven (Figure 5). Macroscopically, a small cleavage was seen proximal to the footprint over the epiphyseal line (Figure 6). On palpation, no movement could be detected between the epiphysis and metaphysis after failure.

### Lateral load

**Figure 7:**
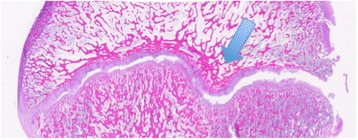
**The epiphyseal tubercle (arrow) pressing against the medial wall of the metaphyseal socket.** The PFC is damaged. Sample stained with hematoxylin-eosin solution. Scanned slide, no magnification.

Imposing a lateral load on all three specimens resulted in failure at a median load of 920 N (range 918–1,148). On histological examination, subtotal epiphysiolysis was found in all specimens, with the damage wandering erratically between the resting and ossifying zones (Figure 4). On those slices in which the epiphyseal tubercle was present, it appeared as though it was partially dislodged and was pressing against the medial wall of the metaphyseal socket (Figure 7). PFC integrity could not be assessed in two slices and the PFC was damaged on eight of the remaining ten slices. Macroscopically, a small cleavage was seen proximal to the footprint, over the epiphyseal line. On palpation, no movement could be detected between the epiphysis and metaphysis.

### Vertical load

Imposing a vertical load on all three specimens resulted in failure at a median load of 7,397 N (range 7,008-7,413). On histological examination, all specimens showed signs, to a varying degree, of epiphyseolysis and fracture epiphyseolysis Salter Harris type 2. The epiphyseolysis was primarily in the ossifying zone. The epiphyseal tubercle was present in one slice in every specimen and the fractures tended to originate laterally to the epiphyseal tubercle and extend through the medial cortex of the femoral neck. The integrity of the PFC could not be assessed in six slices, but the PFC was damaged in three of the remaining six slices. Macroscopically, a cleavage was seen laterally over the epiphyseal line and an obvious fracture, medially on the femoral neck, was seen. On palpation, movement over the fracture could easily be detected.

### General

Loading of all the specimens (9 of 9) resulted in an epiphyseolysis (anterior load and lateral load) or a fracture epiphyseolysis. The epiphyseal plate showed the greatest strength in response to vertical loading (mean 7,273 N (SD 229)) and the least strength in response to lateral loading (mean 995 N (SD 132)), with the strength in response to anterior loading falling in between (mean 1,728 N (SD 43)).

**Figure 8:**
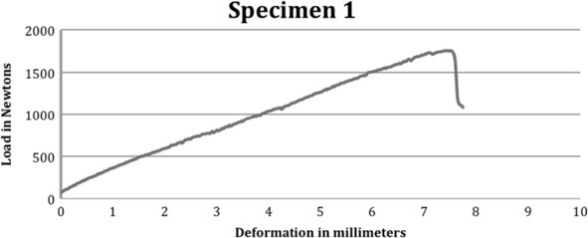
**Anterior loaded specimen sample load/deformation curve.**

The femoral heads were of similar size and the plastic cup on the rod left a shallow footprint on the femoral head after loading. The size of the indentation from the plastic cup was clearly detectable on the femoral head and the size of the footprint was related to the load – larger footprints at higher load failures. The macroscopic characteristics of the porcine hips, the footprint size and distance from the physeal line and the load and deformation at failure are presented in Table 1. A sample loading/deformation curve is presented in Figure 8.

**Table 1:** **Macroscopic characteristics with load and deformation at failure for all specimens**

**Direction of load**	**Specimen**	**AP diameter (mm)**	**ML diameter (mm)**	**Load at failure (N)**	**Deformation at failure (mm)**	**Footprint (mm)**	**Footprint distance from epiphyseal line (mm)**	**Macroscopic epiphyseolysis**
Anterior load								
	1	35	36	1756	7.4	22 × 8	6	Yes
	2	35	37	1750	7.5	21 × 7	5	Yes
	3	35	36	1679	8.7	20 × 8	5	Yes
	Mean (SD)	35	36	1728 (43)	7.9			
Lateral load								
	1	34	36	1148	7.1	14 × 8	4	Yes
	2	34	36	918	7.0	15 × 4	3	Yes
	3	37	38	920	7.1	17 × 4	4	Yes
	Mean (SD)	35	37	995 (132)	7.1			
Vertical load								
	1	38	39	7397	5.0	25 × 25	5	Yes
	2	37	39	7413	6.6	25 × 25	9	Yes
	3	38	38	7008	6.4	25 × 25	4	Yes
	Mean (SD)	38	39	7273 (229)	6.0

On nine of thirty-six slices, PFC integrity could not be estimated. During preparation of the slices, the metaphyseal part of the PFC disappeared, making judgement of its integrity impossible. The PFC was damaged on 12 of the remaining 27 slices. All specimens loaded laterally showed some damage to the PFC. The epiphyseal tubercle was identified on 12 of 36 slices. Its presence was confirmed in all specimens apart from one loaded anteriorly. The microscopic characteristics are presented in Table 2.

**Table 2:** **Microscopic characteristics of the specimens**

**Specimen**	**Slide**	**Microscopic epiphyseolysis present**	**Percentage of epiphysis damaged**	**Epiphyseal zone damaged**	**Salter Harris classification**	**Perichondral fibrocartilaginous complex damaged**	**Epiphyseal tubercle present**
Anterior specimen 1							
	1	Yes	100	Resting/ossifying	1	No	No
	2	Yes	68	Resting	1	No	No
	3	Yes	77	Resting/ossifying	1	No	No
	4	Yes	100	Resting/ossifying	2	Yes	Yes
Anterior specimen 2							
	1	Yes	89	Resting/ossifying	2	No	No
	2	Yes	63	Ossifying	1	No	No
	3	Yes	80	Resting/ossifying	1	N/A	No
	4	Yes	81	Resting/ossifying	1	No	No
Anterior specimen 3							
	1	Yes	100	Resting/ossifying	1	No	No
	2	Yes	100	Resting/ossifying	1	No	No
	3	Yes	100	Resting/ossifying	1	No	Yes
	4	Yes	100	Resting/ossifying	1	No	Yes
Lateral specimen 1							
	1	Yes	83	Resting/ossifying	1	Yes	No
	2	Yes	84	Ossifying	1	No	No
	3	Yes	87	Resting/ossifying	1	N/A	Yes
	4	Yes	100	Resting/ossifying	2	No	Yes
Lateral specimen 2							
	1	Yes	100	Resting/ossifying	2	Yes	No
	2	Yes	55	Resting/ossifying	1	Yes	No
	3	Yes	75	Resting/ossifying	1	Yes	Yes
	4	Yes	75	Resting/ossifying	1	Yes	Yes
Lateral specimen 3							
	1	Yes	64	Resting/ossifying	1	Yes	No
	2	Yes	100	Resting/ossifying	2	N/A	No
	3	Yes	78	Resting/ossifying	1	Yes	Yes
	4	Yes	72	Resting/ossifying	1	Yes	Yes
Vertical specimen 1							
	1	Yes	100	Ossifying	1	No	No
	2	Yes	50	Ossifying	1	No	No
	3	Yes	30	Ossifying	1	N/A	Yes
	4	Yes fracture	30	Resting	2	N/A	No
Vertical specimen 2							
	1	Yes fracture	70	Ossifying	2	Yes	No
	2	Yes fracture	80	Ossifying	2	Yes	No
	3	Yes fracture	100	Ossifying	2	N/A	Yes
	4	Yes fracture	50	Ossifying	2	Yes	No
Vertical specimen 3							
	1	Yes fracture	80	Ossifying	2	No	No
	2	Yes fracture	100	Ossifying	2	N/A	No
	3	Yes fracture	100	Ossifying	2	N/A	Yes
	4	Yes fracture	100	Ossifying	2	N/A	No

The force/deformation graphs for lateral, anterior and vertical loads are presented in Figures 2, 3 and 4 respectively.

## Discussion

The main finding in the present study is that the epiphyseal plate is the weakest point in the young porcine proximal femur. Loading to failure resulted in epiphyseolysis or fracture epiphyseolysis in all the specimens. The model was consistent and easy to set up.

Load at failure was lowest for the laterally loaded specimens (995 N) and highest for the vertically loaded specimens (7,273 N). The load set-up in the present model resulted primarily in a tensile load over the epiphyseal plate in the laterally loaded specimens, a shear load in the anteriorly loaded specimens and a compressive load in those loaded vertically. The highest strength was therefore to be expected in the vertically loaded specimens, where the loading direction is comparable to the loading direction *in vivo* [18–21].

The vertical load mainly caused failure in the ossifying zone of the epiphyseal plate, while a more random pattern of failure between different epiphyseal zones was seen in the other groups. In a study of cattle, Moen et al. reported that compressive forces tended to cause failure in the ossifying zone [23].

**Figure 9:**
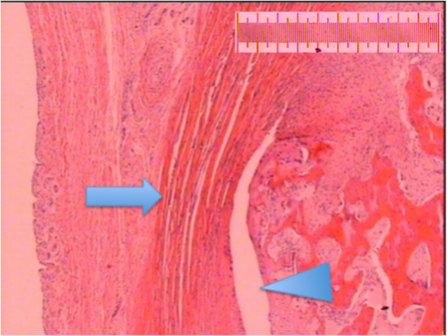
**Photomicrograph showing microscopic epiphyseolysis (arrowhead) with the PFC (arrow) intact.** Sample stained with hematoxylin-eosin solution. Magnification X20. Scale bar equals 1 mm.

Chung et al. [12] reported a significant decrease in shear strength when the PFC was excised. They loaded their specimens anteriorly. In the present study, all but one slice from the anteriorly loaded specimens showed an undamaged PFC (figure 9), while some PFC damage was seen in all laterally loaded specimens. This indicates that the PFC performs a stabilising function, at least when the epiphysis is loaded anteriorly. In the vertically loaded specimens, the integrity of the PFC could not be assessed in six of 12 slices. The disappearance of the metaphyseal part during the preparation of the slices might indicate that the PFC was damaged.

The epiphyseal tubercle was identified in all specimens apart from one of the anteriorly loaded specimens. On the slices from the laterally loaded specimens where the tubercle was identified, the tubercle can be seen pressing against the metaphyseal socket, stopping the slide of the epiphysis (Figure 7). The same finding was seen on the slices from the vertically loaded specimens, where the tubercle appears to stop the epiphysis in its slide. Due to the compressive forces applied in the vertically loaded specimens, the tubercle compresses the medial wall of the socket, resulting in a fracture of the metaphysis through the medial cortex instead of dislodging the tubercle from the socket. This implies that the tubercle performs a stabilising function during vertical and lateral loading.

On microscopic examination of the vertically loaded specimens, the epiphyseal plate was damaged laterally to the epiphyseal tubercle. The cam deformity is usually located antero-superiorly on the femoral head [24] and therefore laterally to the epiphyseal tubercle. It is possible to speculate that the cam deformity develops from a partial epiphyseolysis, where the epiphyseal tubercle prevents total epiphyseolysis or dislodges partially in total epiphyseolysis. Rotation of the epiphysis around the eccentric epiphyseal tubercle has also been suggested as a possible cause of chronic SCFE [16,17].

Anterior and lateral loading forces around the femoral head are small during routine activities [18–21]. The way loading occurs during sports and in deep flexion is unknown. Muscle action and ligament constraints around the hip joint most probably prevent the joint being loaded from an anterior direction *in vivo*. In reality, the hip joint is probably loaded somewhere between the vertical and anterior loading directions in deep flexion. A posterior slope of the femoral epiphysis increases the anterior load on the epiphyseal plate and is a known predictor of SCFE [25] and anterior load also appears to increase in deep flexion [26]. It is possible to speculate that the strength of the epiphyseal plate is therefore lower when the hip is in flexion.

### Limitations

Using an *in-vitro* porcine model can be regarded as a limitation when it comes to understanding human hip joint diseases, but many studies of hip biomechanics and hip conditions use a porcine model, especially in pediatric orthopedics, [27–34] and, in a phenomenological study like the present one, it is thought to be appropriate.

During the preparation of the specimens, all the soft tissues were removed. The muscles and ligaments of the hip most certainly play a major role in dynamic hip biomechanics. Our conclusions are not affected by this fact, as we are only investigating semi-static strength.

**Figure 10:**
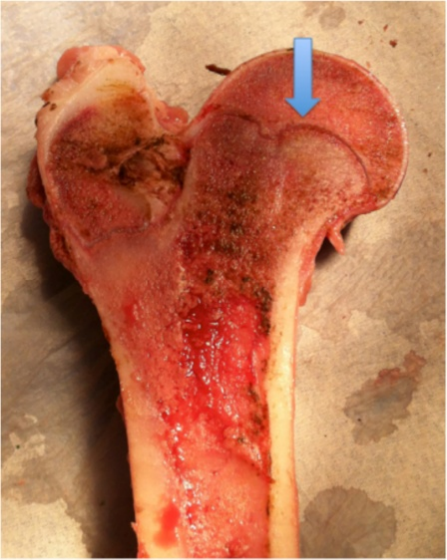
**Porcine proximal femur split coronally.** The major trochanter is relatively larger than in a human and the femoral neck shorter. The growth plate and the epiphyseal tubercle are clearly visible in this specimen (arrow).

**Figure 11:**
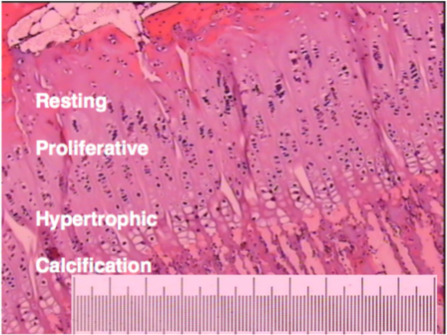
**The different zones of the porcine epiphyseal plate.** Sample stained with hematoxylin-eosin solution. Magnification X40. Scale bar equals 1 mm.

Macroscopically, the porcine hip is comparable to the human hip, the main difference being a more prominent major trochanter and a shorter femoral neck (Figure 10). Ipsen et al. reported that, in one-year-old pigs, the proximal femoral epiphysis is similar in size to that in adolescent humans and its shear strength is comparable to that reported by Chung et al. [12,35]. The specimens in the present study are of comparable size to those in a 10-year-old child and the shear strength of the anteriorly loaded specimens was comparable to the values reported by Chung et al. [12]. The microscopic characteristics of the porcine epiphyseal plate are identical to those of the human epiphyseal plate, with the different epiphyseal growth zones clearly visible (Figure 11).

## Conclusion

This model is consistent and simple. The weakest part in the young porcine proximal femur was the epiphyseal plate. This location of weakness may explain the cam deformity found in young athletes. The epiphyseal plate is strongest when exposed to vertical load, weaker when exposed to anterior load and weakest when exposed to lateral load. The epiphyseal tubercle and the PFC are important stabilisers of the epiphysis.
